# Efficacy of acupuncture-related therapy for postmenopausal osteoporosis: a systematic review and network meta-analysis based on randomized controlled trials

**DOI:** 10.3389/fmed.2025.1483819

**Published:** 2025-04-09

**Authors:** Bing Deng, Tiantian Xu, Zilan Deng, Yue Jiang, Li Li, Wankun Liang, Yuewen Zhang, Hongjin Wang, Yunxiang Xu, Guizhen Chen

**Affiliations:** ^1^Shenzhen Bao’an Traditional Chinese Medicine Hospital, Guangzhou University of Chinese Medicine, Shenzhen, China; ^2^Clinical Medical College of Acupuncture-Moxibustion and Rehabilitation, Guangzhou University of Chinese Medicine, Guangzhou, China

**Keywords:** postmenopausal osteoporosis, acupuncture, the overall clinical effectiveness rate, systematic review, network meta-analysis

## Abstract

**Introduction:**

To compare and analyze the clinical effects of acupuncture-related therapies for postmenopausal osteoporosis (PMOP) and propose the optimal scheme, we utilized a network meta-analysis to evaluate the therapeutic effects of various commonly used acupuncture methods for PMOP.

**Methods:**

Randomized controlled trials of acupuncture-related therapies for PMOP were searched in eight databases (PubMed, Embase, Cochrane Library, Web of Science, China National Knowledge Infrastructure, China Science and Technology Journal Database, China Biomedical Literature Database, and Wanfang database) from January 1, 2002 to December 31, 2023. Our primary outcomes included overall clinical effectiveness rate, bone mineral density (BMD), and visual analog scale scores (VAS). The secondary outcome is adverse events. The entire process of literature screening and data analysis was conducted by 2 independent investigators.

**Results:**

A total of 30 studies with 2,342 participants provided data suitable for analysis. We compared six interventions: manual acupuncture, electroacupuncture, acupoint catgut embedding, moxibustion, acupoint application, and warm acupuncture. The results of the network meta-analysis revealed that, when compared to conventional Western medication (CWM), multiple acupuncture therapies had a greater impact on the overall clinical effectiveness rate. Electroacupuncture combined with CWM demonstrated superior clinical effectiveness and lumbar spine BMD improvement. Moxibustion with CWM ranked highest for femoral neck BMD, while warm acupuncture showed optimal effects on Ward’s triangle and trochanter BMD. Acupoint catgut embedding provided the greatest pain reduction. The most prevalent minor adverse effects included hematoma, discomfort, and scorching.

**Conclusion:**

The results suggest that several acupuncture-related therapies, either alone or in conjunction with CWM, outperform CWM alone and may be regarded as an alternative or supplementary therapy to PMOP, though higher-quality trials are needed.

## 1 Introduction

Osteoporosis is an urgent public health threat characterized by a systemic decrease in bone mass, strength, and microarchitecture, which significantly increase the risk of fragility fractures (1, 2). Epidemiological reports highlight that osteoporosis has become a critical public health concern across Asia, Europe, and the United States due to its high prevalence, complex complications, and substantial economic burden (3–5). Among individuals over the age of 50, approximately 50% of women and 20% of men will suffer osteoporosis-related fractures (6, 7). Postmenopausal osteoporosis (PMOP) is the most prevalent form of osteoporosis and the bone disease most frequently observed in postmenopausal women (8, 9). With the loss of bone mass and the destruction of bone microstructure, patients with PMOP may experience symptoms such as back and leg pain, limb weakness, hunchback, and an increased susceptibility to fractures (10, 11). As global populations age rapidly, PMOP is poised to become a worldwide challenge, adversely affecting physical and mental health while imposing unsustainable societal costs (12–14). Current PMOP management strategies include lifestyle modifications, pharmacological interventions, and rehabilitation therapies (15). The most commonly employed anti-fracture strategies for PMOP involve a combination of calcium and vitamin D, hormone replacement therapy (HRT), bisphosphonates, and selective estrogen receptor modulators (SERMs), among others (13, 16). However, we cannot overlook the adverse effects and limited efficacy of the currently used medications, including vaginal bleeding, breast tenderness, deep vein thrombosis, and cardiovascular events. Therefore, there is an urgent need to explore better complementary and alternative therapies for PMOP.

Acupuncture, a traditional Chinese medicine practice with millennia of historical use, has been endorsed by the World Health Organization (WHO) for managing musculoskeletal, neurological, gynecological, and pain-related conditions (17, 18). Evidence-based medical research confirms that acupuncture-related treatments can improve patient’s quality of life, regulate bone metabolism, and reduce pain through holistic adjustments (19–22). Therefore, we selected the overall clinical effectiveness rate, bone mineral density (BMD), and visual analog scale scores (VAS) as outcome indicators, with concurrent safety assessments via adverse reaction. Despite the growing adoption of diverse acupuncture-related therapies for PMOP, clinical practitioners face challenges in selecting optimal modalities due to insufficient comparative evidence. Therefore, we used network meta-analysis (NMA) to compare the efficacy of various acupuncture-related therapies across different outcome indicators, with the expectation of providing a reliable evidence-based medical basis for the promotion and evaluation of clinical acupuncture for PMOP.

Compared to previous systematic reviews and paired meta-analysis (19, 21, 22), NMA can simultaneously evaluate both direct and indirect evidence from various studies and estimate the relative effectiveness of multiple acupuncture therapies. To our knowledge, this is the first NMA to compare and rank acupuncture-related interventions for PMOP, identify the most effective clinical protocols, and inform evidence-based guidelines.

## 2 Methods

The article adhered to the Preferred Reporting Items for Systematic Review and Meta Analysis (PRISMA) guidelines for NMA, as detailed in Supplementary Appendix 1 (23). The review was registered in PROSPERO^1^ with the registration ID of CRD42023401003. As this is a systematic literature review, ethical approval is not required. The protocol was published in *BMJ OPEN* (24).

### 2.1 Search strategy

We conducted a systematic review of randomized controlled trials (RCTs) investigating acupuncture-related therapies for PMOP across eight databases: PubMed, Embase, Cochrane Library, Web of Science, China National Knowledge Infrastructure (CNKI), China Science and Technology Journal Database (VIP), Wanfang Database, and China Biomedical Literature Database (CBM). We searched each database from January 1, 2002, to December 31, 2023, and the language was restricted to Chinese and English with a combination of Medical Subject Headings and free words. The search terms included “acupuncture” “electroacupuncture” “moxibustion” “warm needle acupuncture” “catgut implantation at acupoint” “postmenopausal osteoporosis” and their synonyms (Supplementary Appendix 2).

### 2.2 Inclusion criteria

Criteria for study inclusion: (1) Randomized controlled trials (RCTs) investigating various acupuncture-related methods for the treatment of PMOP, published in English or Chinese; (2) The study population consisted of patients with a clinical diagnosis of PMOP, adhering to the relevant diagnostic criteria established by the European guidelines for diagnosis and management in 2018 and the 2021 position statement of The North American Menopause Society (25); (3) Patients in the treatment group accepted acupuncture-related therapies, including acupuncture, warm needle, electro-acupuncture, fire needle, blood-letting puncture, moxibustion, acupoint catgut embedding, acupoint application, and more (26–28). The control group contained placebo, usual care, and CWM. When the treatment group was to be treated with a combination of CWM, it was ensured that these should be identical in control groups. (4) Study outcomes included the overall clinical effectiveness rate, BMD, VAS, and adverse events. We mainly incorporated the lumbar spine, femur neck, ward’s triangle, and trochanter (G.T.) for BMD. The overall clinical effectiveness rate, based on the criteria of Chinese medicine clinical evidence points for the clinical standard used to judge the efficacy of acupuncture-related therapies, will be divided into four levels: clinically cured, markedly effective, effective, and invalid (21, 29). The overall clinical effectiveness rate will be calculated as: the overall clinical effectiveness rate (%) = [(number of patients clinically cured + markedly effective + effective)/number of patients] × 100% (30).

### 2.3 Exclusion criteria

Criteria for study exclusion: (1) Non-RCT studies, animal or basic studies; (2) Comorbidities with other diseases that may influence the assessment of efficacy, such as breast disease, thyroid dysfunction, insomnia, anxiety, and depression; (3) Interventions in which the control group received non-conventional Western medicine or two types of acupuncture therapies simultaneously, along with unclear descriptions of the interventions; (4) Unavailability of raw data.

### 2.4 Study selection and data extraction

Two reviewers utilized EndNote software to eliminate duplicate documents, and then independently screened all abstracts and full papers. Any disagreements were resolved through discussion or by consulting a third reviewer. The reviewers collected detailed data including study characteristics (author, publication year, mean age, sample size), intervention and control measures (acupoints, operation, treatment duration, frequency), diagnostic criteria, and outcomes. Each evaluator cross-checked the selected studies, extracted data, and resolved any disagreements by consulting a third party.

### 2.5 Risk of bias assessment

Two reviewers (BD and TTX) independently assessed the risk of bias in the included studies according to the Cochrane Manual’s risk of bias assessment tool for RCTs. The evaluation encompassed 7 items: random sequence generation, assignment plan concealment, blinding of participants and personnel, blinding of study outcome measures, the integrity of outcome data, selective outcome reporting, and other sources of bias. Each domain was categorized as “low,” “high,” and “uncertain.” In cases of disagreement, a third researcher (GZC) was consulted to help determine the risk.

### 2.6 Statistical analysis

We performed Bayesian NMA to compare the effects of different acupuncture-related therapies. We primarily performed a pairwise meta-analysis using the software Review Manager V.5.4 and Stata 14.0. The heterogeneity of each pairwise comparison was assessed using the Q test and the *I^2^* statistic through RevMan V.5.4. The effect values of 95% Confidence Intervals (CIs) were measured by the software of Stata 14.0. Since the overall clinical effectiveness rate is a dichotomous type variable, the number of events and the total sample size were utilized as the effective values for these dichotomous type variables in the statistical analyses, reported using odds ratio (OR) with 95% Confidence Intervals. The BMD and VAS, which are continuous type variables, were expressed as mean difference (MD) and 95% confidence intervals.

Additionally, we employed Stata 14.0 for data analysis and graph drawing. The “sucra prob” command was used to rank the efficacy of different interventions and to create a cumulative probability graph. The surface under the cumulative ranking curve (SUCRA) indicated the relative superiority or inferiority of each intervention; a larger SUCRA value signifies better efficacy for the outcome in question. *p* < 0.05 was considered statistically significant. We generated funnel plots using stata’s NMA package to evaluate publication bias and the small sample size of the included literature. We conducted a narrative review and summarised the evidence if the available data were not suitable for synthesis.

The *I^2^* statistic and *p* values were applied to assess the heterogeneity across all individual studies. To obtain more reliable estimates of the effect, *I^2^* greater than or equal to 50% and *p* < 0.1 were used as thresholds to indicate significant heterogeneity. If the heterogeneity is small, we chose the fixed-effects model. Based on the information we collected, subgroup analyses of bone density were performed at different sites, including the bone density of the lumbar spine, femoral neck, ward’s triangle, and trochanter.

## 3 Results

### 3.1 Literature search and screening

**Figure 1:**
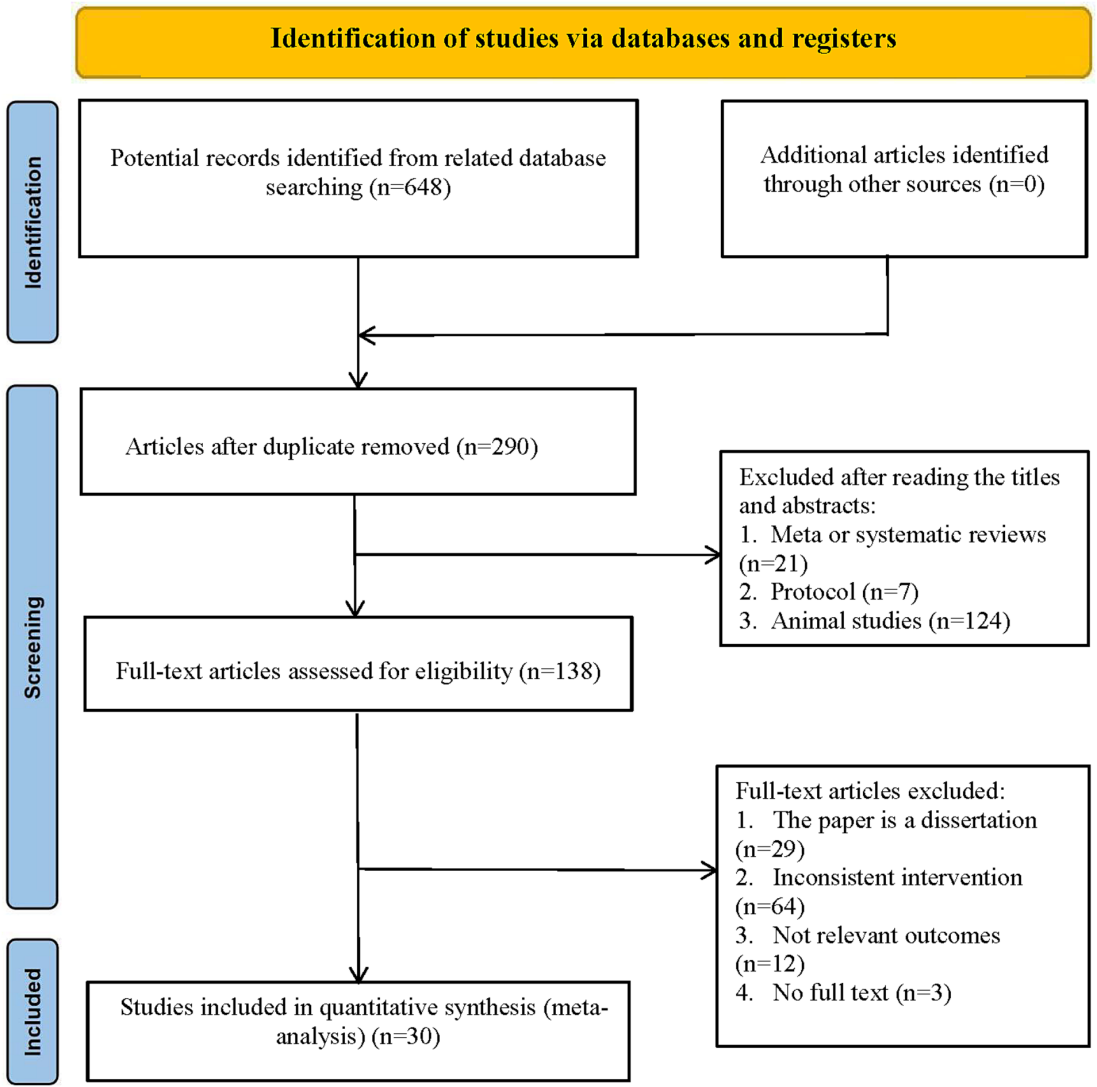
Preferred reporting items for systematic reviews and network meta-analyses (PRISMA) flowchart of the literature inclusion process.

A total of 648 potentially relevant articles were identified, of which 358 were duplicate records. Additionally, 152 articles that were literature reviews, systematic reviews, or animal experiments were further excluded. After reviewing the titles, abstracts, and full texts, we found that 108 articles were ineligible for inclusion because they were not RCTs, or because the studies or interventions did not align with our criteria. Ultimately, we included 30 eligible studies (31–60). The PRISMA flow diagram illustrating the study inclusion process is presented in Figure 1.

### 3.2 Study characteristics

These studies all were from China and were published between 2004 and 2022. A total of 2,342 participants were involved in these studies. Among 30 RCTs, four studies were three arms and twenty-six studies were two-arm trials. The acupuncture-related therapeutic interventions in the treatment groups included electroacupuncture (EA) (31–33), moxibustion (MO) (34–38), warm acupuncture (WA) (31, 39–44), acupoint application (AA) (45, 46), acupoint catgut embedding (ACE) (47–54), and manual acupuncture (MA) (55–60). The control groups all utilized oral Western medications for the treatment of PMOP. The majority opted for a combination of Calcium Carbonate and Vitamin D or used either alone, and only one study chose estradiol as the control (60). The basic characteristics of the included studies are shown in Table 1 and acupuncture-related therapies are described in more detail in Table 2.

**Table 1:** Characteristics of included studies.

Author (year)	Sample size			Mean age (years) (T/C)			Interventions			Acupoints	Course of treatment	Outcomes
	T1	T2	C	T1	T2	C	T1	T2	C			
Chen et al. (2004) (39)	30	-	30	58.12 ± 7.25	-	57.63 ± 7.68	WA + CC + VD	-	CC + VD	DU14, ST36, BL23, BL26	6 M	①②
Wang et al. (2004) (55)	50	-	50	47.87	-	49.62	MA	-	CC	BL23, BL18, ST36, KI1, SP10	3 M	①②
Lin et al. (2005) (47)	18	20	18	60.06	62.55	60.83	ACE	ACE+CC + VD	CC + VD	BL23	3 M	①③
Lin et al. (2006) (48)	18	20	18	60.06 ± 6.12	62.55 ± 5.91	60.83 ± 5.65	ACE	ACE+CC + VD	CC + VD	BL23	6 M	②③
Gao et al. (2008) (56)	30	-	28	63.50 ± 3.85	-	63.50 ± 3.85	MA	-	CC + VD	RN13, RN12, RN6, RN4, ST25	3 M	①
Zhao et al. (2008) (44)	20	-	20	64.00 ± 5.99	-	66.90 ± 5.89	WA	-	CC + VD	BL11, BL18, BL23, ST36, GB34	3 M	①②
Wang (2009) (60)	56	-	56	55.3	-	55.3	MA	-	E2	BL23, GV4, CV4	3 M	①
Du (2011) (57)	32	-	32	64 ± 8	-	66 ± 9	MA	-	CC + VD	LR4, BL18, BL23, BL20, ST36, GB34, GB39, SP6, KI3, LR3	3 M	①②
Liu et al. (2011) (49)	45	-	45	62.5 ± 9.7	-	59.8 ± 8.6	ACE+CC + VD	-	CC + VD	BL23, BL18, BL20, EX-B2, BL40	6 M	①②③④
Liu et al. (2011) (49)	35	-	35	63.7 ± 3.8	-	62.8 ± 5.9	ACE+CC + VD	-	CC + VD	BL23, BL18, EX-B2, BL40	6 M	①②③④
Zhou et al. (2012) (32)	50	-	50	58 ± 5	-	56 ± 7	EA + VD	-	VD	BL20, BL21, BL23, BL18, DU3, DU4, RN12, ST25, RN4, ST36, SP6, KI3, GB39	6 M	①②③④
Liang et al. (2013) (51)	60	-	60	65.7 ± 6.9	-	66.9 ± 5.8	ACE+CC + VD	-	CC + VD	BL23, BL18, EX-B2, BL40	6 M	①②③④
Lin et al. (2013) (34)	32	-	31	-	-	-	MO + CC + VD	-	CC + VD	From DU2 to DU14	3 M	③
OuYang and Xu (2013) (35)	29	-	28	62.45 ± 7.68	-	62.45 ± 7.68	MO + CC + VD	-	CC + VD	BL20, BL21, BL23, DU4, DU3, DU9	3 M	①
Wang et al. (2013) (33)	50	50	50	-	-	-	EA + VD	EA	VD	BL20, BL21，BL18, BL23, RN12, RN4, ST36, SP6	6 M	①②③
Cai et al. (2014) (31)	30	30	30	51 ± 6	52 ± 7	50 ± 6	WA	EA	CC + VD	BL11, BL23, GB39	1 M	③
Lu (2014) (52)	25	-	22	60.84 ± 6.956	-	62.14 ± 6.342	ACE+CC + VD	-	CC + VD	BL23	6 M	①②③
Cai et al. (2015) (40)	43	-	42	51 ± 7	-	50 ± 6	WA + CC + VD	-	CC + VD	BL11, BL23, GB39	1Y	②
Yu (2015) (36)	20	-	20	62.27 ± 8.73	-	62.01 ± 0.73	MO + CC + VD	-	CC + VD	DU4, BL23	1Y	①②
Luo (2015) (41)	18	-	18	54.8 ± 6.2	-	54.8 ± 6.2	WA	-	CC + VD	BL18, BL23, GB34, ST36, BL11, SP6, GB39, RN4	6 M	①②
He et al. (2015) (45)	85	-	91	-	-	-	AA+VD	-	CC + VD	DU4, BL23, BL22, BL52, RN6, RN4, DU3, BL24, BL26	6 M	②③
Hu (2016) (58)	32	-	35	61.4 ± 8.3	-	62.1 ± 8.7	MA + CC + VD	-	CC + VD	BL20, BL21, BL23, BL18, ST36	3 M	①②
Chai and Liang (2017) (42)	33	-	32	50. 26 ± 5. 91	-	49. 85 ± 5. 69	WA + CC + VD	-	CC + VD	BL23, DU4, RN4, RN6	3 M	①②
Li (2017) (43)	60	-	60	65.35 ± 5.73	-	66.17 ± 5.65	WA	-	CC + VD	BL18, BL23, GB34, ST36, BL11, RN4, SP6	6 M	①③
Gu et al. (2018) (54)	30	-	30	62 ± 5	-	62 ± 5	ACE+VD	-	VD	stellate ganglion	24 W	①②
Wu et al. (2018) (37)	28	-	28	59.25 ± 2.91	-	58.04 ± 3.20	MO + CC + VD	-	CC + VD	RN8	16 W	②③
Lin et al. (2020) (38)	30	-	30	59.50 ± 5.92	-	60.75 ± 5.09	MO + CC + VD	-	CC + VD	From DU2 to DU14	3 M	③
Yang et al. (2021) (53)	58	-	52	57.3 ± 3.5	-	56.2 ± 3.7	ACE	-	CC + VD	CV4, RN6, BL23, SP6, GB30, ST36	24 W	①④
Chen et al. (2022) (59)	31	-	32	64 ± 5	-	64 ± 5	MA + CC + VD	-	CC + VD	BL23, BL20, RN4, ST36, GB39, SP6	12w	①②④
Li et al. (2022) (72)	50	-	51	57.92 ± 3.46	-	56.63 ± 3.19	AA+CC + VD	-	CC + VD	BL20, BL23, RN8, KI1, RN4	6 M	②

T: Intervention of treatment; C: Intervention of control; Interventions: EA, electroacupuncture; MO, moxibustion; WA, warm acupuncture; AA, acupoint application; ACE, acupoint catgut embedding; MA, manual acupuncture; CC, Calcium Carbonate; VD, Vitamin D; E2, estradiol; Course of treatment: W, weeks; M, months; Y, years; Outcomes: ① The overall clinical effectiveness rate, ② The lumbar spine bone mineral density, ③ Visual analog scale score, ④ Adverse events.

**Table 2:** Definitions of the acupuncture-related therapies included in this study.

Acupuncture-related therapy	Definitions
Manual acupuncture (MA)	Manual acupuncture involves the insertion of fine needles into acupuncture points for treatment. The needles are usually manipulated by a physician with the aim of eliciting the Deqi sensation.
Electroacupuncture (EA)	After Deqi by inserting the acupoint with a needle, a trace current wave of the body’s bioelectricity is sensed on the needle.
Moxibustion (MO)	It is the use of moxa leaves made of moxa sticks, moxa pillars, produced by the heat of moxa to stimulate the human body acupuncture points or specific areas.
Acupoint catgut embedding (ACE)	Catguts are embedded into specific acupoints by means of needles to produce long-lasting stimulation of the acupoints to achieve the therapeutic effect.
Acupoint application (AA)	A combination of acupoint and medicine. The medicine is applied directly to the acupoints and stimulated by transdermal absorption through the skin at the acupoints.
Warm acupuncture (WA)	A combination of acupuncture and moxibustion. During the process of needle retention, moxa floss is rolled and twisted and wrapped around the handle of the needle and ignited, and the heat is transmitted into the acupuncture point through the body of the needle.

### 3.3 Study quality assessment

**Figure 2:**
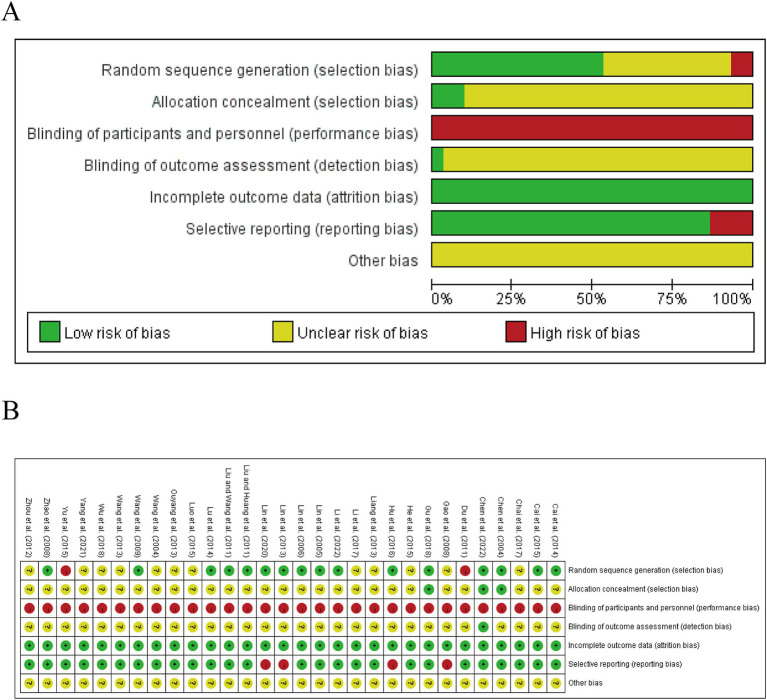
The summary chart **(A)** and detailed graph **(B)** for the bias risk assessment.

Thirteen studies randomized participants using a random number table (31, 34, 38–40, 44, 46–50, 58, 60), while one study employed Doll’s clinical case randomization table grouping method (52), two studies were grouped by the order of patient visits in a non-randomized manner (36, 57). Three studies utilized sealed opaque envelopes for allocation concealment (39, 54, 59), whereas the remaining twenty-seven studies made no mention of their randomization procedures. Because of the specificity of acupuncture-related therapies, blinding of participants and personnel requires a specialized design, such as the use of sham acupuncture devices in the control group. However, this was not specified in any of the studies we included. Regarding detection bias, only one study has been described (59). No other biases were detected in the included studies. The data integrity of the included literature was good, and other risks of bias were unknown. The results of the risk of bias evaluation are presented in Figures 2A,B.

### 3.4 Network meta-analysis results

#### 3.4.1 The overall clinical effectiveness rate

**Figure 3:**
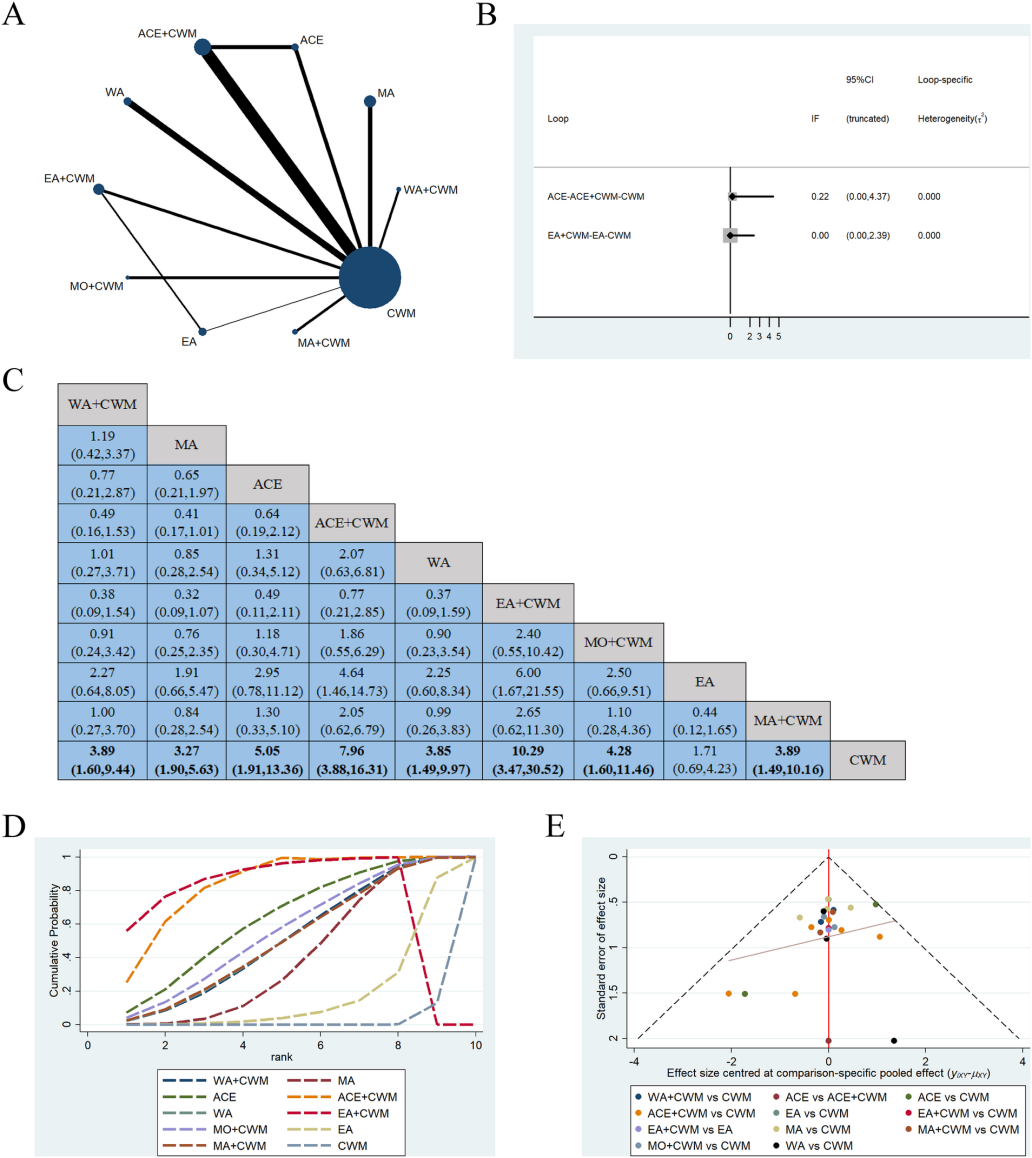
Network meta-analysis of multiple acupuncture-related treatments for clinical effectiveness rate, the line thickness is related to the number of comparisons, and the node size is proportional to the sample size **(A)**. Inconsistency test of the overall clinical effectiveness rate, *p* > 0.05 indicates good consistency **(B)**. The league figure of the incidence rate of improving the clinical effectiveness rate **(C)**. SUCRA value of the overall clinical effectiveness rate of PMOP, a bigger SUCRA value represents better efficacy of the intervention **(D)**. Funnel plot of the overall clinical effectiveness rate for the network meta-analysis, the symmetrical distribution suggests a low risk of publication bias **(E)**. PMOP, postmenopausal osteoporosis.

Twenty-two studies were included in the statistical analysis of the efficacy of treatments for PMOP, with the network relationship between interventions shown in Figure 3A (32, 33, 35, 36, 39, 41–44, 47, 49–60). Line thickness corresponds to the number of studies included in the comparison of each treatment, while the area of the circles indicates the overall sample size associated with each intervention. Depending on the thickness of the line linking, the number of RCTs between ACE+CWM and CWM was the highest. Additionally, the sample size for CWM was the largest, followed by that of ACE+CWM. Due to the formation of two closed loops, the overall clinical effectiveness rate was initially assessed using an inconsistent model (Figure 3B), where the *p* in the ACE-ACE+CWM-CWM and EA–EA + CWM-CWM closed loops were 4.37 and 2.39, suggesting that the inconsistency was not statistically significant.

Figure 3C is about the pairwise comparison results of ten types of interventions with controls. The findings indicated that WA + CWM (OR = 3.89, 95%CI:1.60–9.44), MA (OR = 3.27, 95%CI:1.90–5.63), ACE (OR = 5.05, 95%CI:1.91–13.36), ACE+CWM (OR = 7.96, 95%CI:3.88–16.31), WA (OR = 3.85, 95%CI:1.49–9.97), EA + CWM (OR = 10.29, 95%CI:3.47–30.52), MO + CWM (OR = 4.28, 95%CI:1.60–11.46), and MA + CWM (OR = 3.89, 95%CI:1.49–10.16) had superior efficacy rates for improving postmenopausal osteoporosis compared to the CWM (*p* < 0.05). However, there was no significant difference among the various acupuncture-related treatments. The results of pairwise comparisons and ranking from the reticular meta-analysis were presented in Figure 3D. According to SUCRA values, EA + CWM (89.6%) > ACE+CWM (83.9%) > ACE (63.0%) > MO + CWM (55.2%) > WA + CWM (50.2%) = MA + CWM (50.2%) > WA (50.1%) > MA (40.0%) > EA (16.4%) > CWM (1.5%) the differences were statistically significant (*p* < 0.05). We found that EA + CWM group ranked first as the optimal treatment for PMOP. A comparison-adjusted funnel plot was used to analyze the publication bias of the overall clinical effectiveness rate. The differently colored points in this funnel plot represented different direct comparisons and the number of the same color points indicated the frequency of those comparisons in the original study. In the Figure 3E, most of the points were evenly distributed on both sides of the middle vertical line. The majority of the research included had moderate sample sizes, and the funnel plot suggested that these studies were biased to a low degree.

#### 3.4.2 The BMD

**Figure 4:**
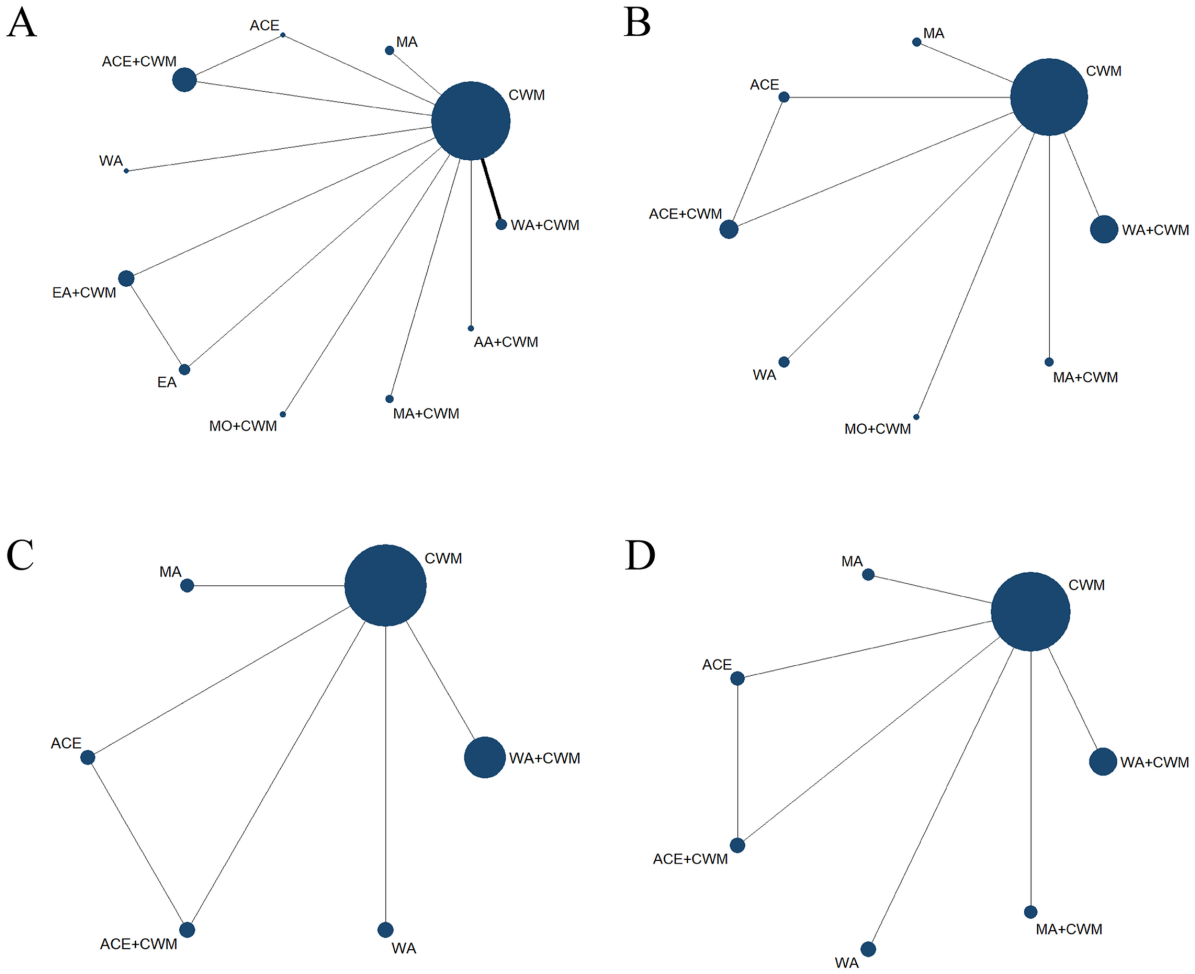
Network diagrams of comparisons of bone density at different sites of treatments in patients with PMOP, the line thickness is related to the number of comparisons, and the node size is proportional to the sample size. Network meta-analysis of multiple acupuncture-related treatments for lumbar spine BMD **(A)**. Network meta-analysis of multiple acupuncture-related treatments for femoral neck BMD **(B)**. Network meta-analysis of multiple acupuncture-related treatments for ward’s triangle BMD **(C)**. Network meta-analysis of multiple acupuncture-related treatments for trochanter BMD **(D)**. BMD, bone mineral density.

**Figure 5:**
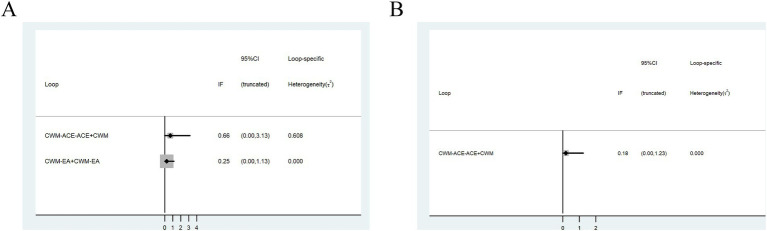
Inconsistency test. Lumbar spine BMD **(A)**; femoral neck bone density **(B)**.

**Figure 6:**
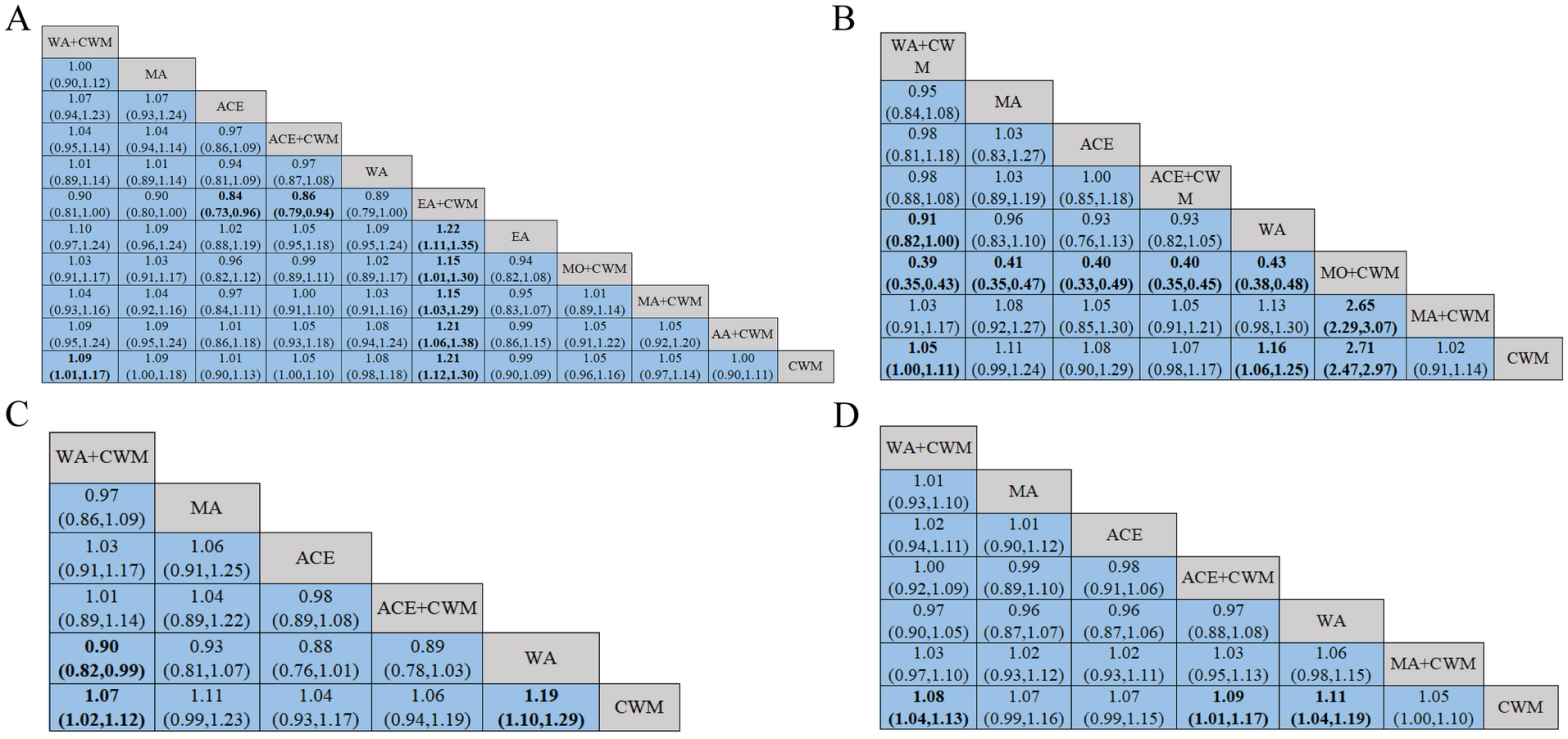
League figure. The bold font indicates a statistical difference. League figure of response rate of lumbar spine BMD **(A)**. League figure of response rate of femoral neck BMD **(B)**. League figure of response rate of ward’s triangle BMD **(C)**. League figure of response rate of trochanter BMD **(D)**. BMD, bone mineral density.

**Figure 7:**
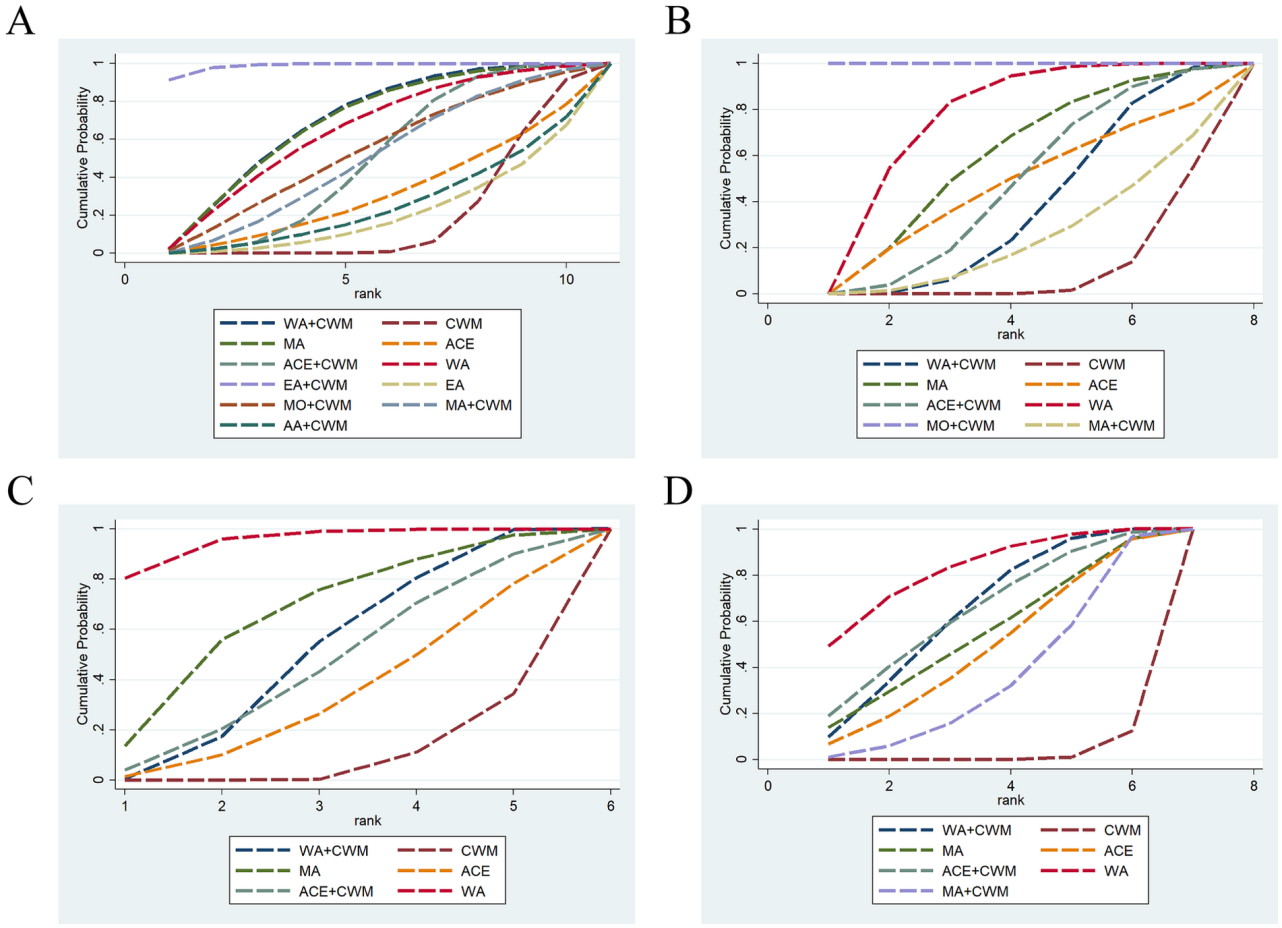
SUCRA value. A bigger SUCRA value represents better efficacy of the intervention. SUCRA value of lumbar spine BMD **(A)**. SUCRA value of femoral neck BMD **(B)**. SUCRA value of ward’s triangle BMD **(C)**. SUCRA value of trochanter BMD **(D)**. BMD, bone mineral density.

21 studies were included in the statistical analysis of the BMD in the treatment of postmenopausal osteoporosis, we focused primarily on the outcomes of the lumbar spine, femoral neck, trochanter, and Ward’s triangle (32, 33, 36, 37, 39–42, 44–46, 48–52, 54, 55, 57–59). The lumbar spine BMD exhibited two closed loops: ACE-ACE+CWM-CWM and EA–EA + CWM-CWM (Figure 4A), while the *p*-values for the two closed-loop inconsistencies were 3.13 and 1.13, indicating that these inconsistencies were not statistically significant (Figure 5A). In the lumbar spine BMD of pairwise comparison results (Figure 6A), WA + CWM (MD = 1.09, 95%CI:1.01–1.17) and EA + CWM (MD = 1.21, 95%CI:1.12–1.30) increased bone density better than conventional Western medicine (*p* < 0.05), and other acupuncture-related therapy did not have statistical differences in increasing bone density. What’s more, EA + CWM was more effective than most other acupuncture-related therapies, such as ACE+CWM, MO + CWM, and MA + CWM, among others. According to SUCRA values in Figure 7A, the order of the acupuncture-related treatments to improve lumbar spine BMD: EA + CWM (98.9%) > WA + CWM (69.4%) > MA (68.7%) > WA (64.3%) > MO + CWM (53.2%) > MA + CWM (49.5%) > ACE+CWM (49.2%) > ACE (31.4%) > AA+CWM (25.5%) > EA (20.9%) > CWM (19.0%) all the differences were statistically significant (*p* < 0.05). EA + CWM was the most favorable intervention for enhancing the lumbar spine BMD.

The network of femoral neck bone density had a closed loop: ACE-ACE+CWM-CWM (Figure 4B). Femoral neck BMD was examined using an inconsistency model, which showed *p* = 1.23, indicating that inconsistency was not significant (Figure 5B). According to pairwise comparison results in Figure 6B, WA (MD = 1.16, 95%CI:1.06–1.25) and MO + CWM (MD = 2.71, 95%CI:2.47–2.97) were more effective than CWM. The probability ranking of SUCRA was as follows (Figure 7B): MO + CWM (100.0%) > WA (75.8%) > MA (58.7%) > ACE+CWM (47.3%) > ACE (46.3%) > WA + CWM (37.4%) > MA + CWM (24.5%) > CWM (10.1%). The ranking results demonstrated that the top intervention to improve femoral neck BMD was MO + CWM.

Both ward’s triangle and trochanter formed a closed loop (Figures 4C,D): ACE-ACE+CWM-CWM, since they were all from the same literature, no inconsistency test was required. According to pairwise comparison results in Figure 6C, WA + CWM (MD = 1.07, 95%CI:1.02–1.12) and WA (MD = 1.19, 95%CI:1.10–1.29) increased ward’s triangle BMD better than CWM. The probability ranking of SUCRA was as follows (Figure 7C): WA (95.1%) > MA (66.1%) > WA + CWM (50.7%) > ACE+CWM (45.7%) >ACE (33.3%) > CWM (9.2%). About trochanter BMD in Figure 6D, WA + CWM (MD = 1.08, 95%CI: 1.04–1.13), ACE+CWM (MD = 1.09, 95%CI:1.01–1.17) and WA (MD = 1.11, 95%CI: 1.04–1.19) improving trochanter BMD of postmenopausal osteoporosis was better than the CWM. The probability ranking of SUCRA was as follows (Figure 7D): WA (82.3%) > ACE+CWM (64.0%) > WA + CWM (63.8%) > MA (54.4%) > ACE (48.1%) > MA + CWM (35.1%) > CWM (2.3%). WA was best for improving BMD in both.

#### 3.4.3 The VAS

**Figure 8:**
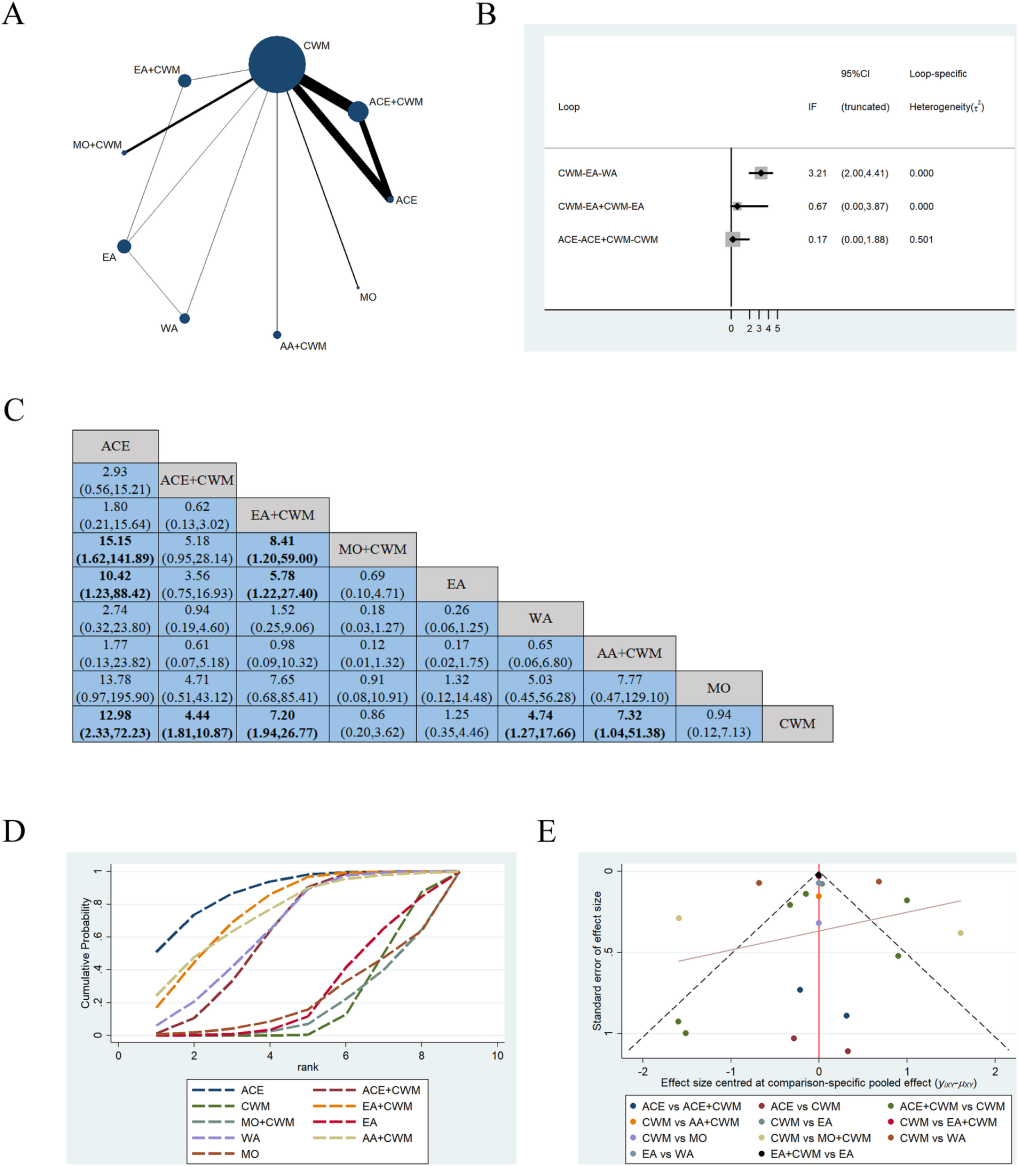
Network meta-analysis of multiple acupuncture-related treatments for VAS, the line thickness is related to the number of comparisons, and the node size is proportional to the sample size **(A)**. Inconsistency test of VAS, *p* > 0.05 indicates good consistency **(B)**. The league figure of the incidence rate of VAS **(C)**. SUCRA value of VAS, a bigger SUCRA value represents better efficacy of the intervention **(D)**. Funnel plot of VAS for the network meta-analysis, the symmetrical distribution suggests a low risk of publication bias **(E)**. VAS, visual analog scale score.

Fourteen studies were included in the statistical analysis of the VAS of postmenopausal osteoporosis treatment (31–34, 37, 38, 43, 45, 47–52). The network relationship between interventions is illustrated in Figure 8A, which shows that the sample size for the ACE+CWM was the largest and the evidence network formed three closed loops. The *p*-value for all three closed loops was greater than 0.05, indicating that this inconsistency was not statistically significant (Figure 8B).

Figure 8C is about the pairwise comparison results of nine types of interventions with controls. In terms of pain relief, ACE (MD = 12.98, 95%CI:2.33–72.23), ACE+CWM (MD = 4.44, 95%CI:1.81–10.87), EA + CWM (MD = 7.20, 95%CI:1.94–26.77), WA (MD = 4.74, 95%CI:1.27–17.66), AA+CWM (MD = 7.32, 95%CI:1.04–51.38) were more efficacious than CWM. Also ACE (MD = 10.42, 95%CI:1.23–88.42) and EA + CWM (MD = 5.78, 95%CI:1.22–27.40) were more efficacious compared to EA. The SUCRA values in Figure 8D, provided a possible rank for improving VAS: ACE (87.9%) > EA + CWM (76.6%) > AA+CWM (74.4%) > WA (64.9%) > ACE+CWM (62.2%) > EA (26.0%) > MO (21.9%) > CWM (18.9%) > MO + CWM (17.2%) the differences were statistically significant (*p* < 0.05), ACE ranked first as the optimal choice. In Figure 8E, the points on the VAS funnel plot are scattered, suggesting a potential publication bias in the research.

#### 3.4.4 Adverse events

Six studies reported the occurrence of adverse events (32, 49–51, 53, 59). One trial involving MA noted that one patient developed a local hematoma at RN4, which dissipated within one week after the application of local hot compresses. Four trials concerning ACE indicated that the treatment group appeared local hard nodules, pain, palpitation, and chest tightness. A study on EA reported adverse effects of stomach pain and insomnia in the acupuncture group. However, these symptoms were mild, and the side effects subsided spontaneously. Some conditions only appeared in the first treatment.

## 4 Discussion

Osteoporosis is the most prevalent skeletal disorder affecting humans and is a widespread bone disease characterized by diminished bone strength. PMOP, caused by ovarian dysfunction and estrogen deficiency in postmenopausal women, severely compromises patients’ quality of life while imposing substantial socioeconomic costs (61, 62). Current first-line pharmacological interventions, though partially effective, are limited by non-negligible adverse effects (63, 64), prompting exploration of complementary and alternative therapies (17, 65, 66). Several basic and clinical studies have confirmed that acupuncture-related therapies have the effect of correcting endocrine metabolic disorders, relieving pain, regulating mental health, and improving quality of life, and may have biological mechanisms such as central sensitization, neurotransmitters, intestinal flora, immune regulation, oxidative stress, and neuroinflammation (67, 68). In this context, as one of the most widely accepted complementary alternative therapies for PMOP, since different acupuncture-related therapies each have their unique advantages, it can be challenging for clinicians to evaluate the therapeutic value of various acupuncture treatments for different conditions (19–22, 69). Therefore, further prospective studies are needed to conduct a comprehensive evaluation of the efficacy of various acupuncture-related therapies for PMOP.

Our analysis incorporated 30 randomized trials (2,342 participants) evaluating six acupuncture modalities. For clinical effectiveness, both direct comparisons and SUCRA rankings identified EA + CWM (89.6%) as the most effective intervention. To evaluate the efficacy of BMD treatments, we performed several subgroup analyses. The first was lumbar spine BMD, the results of which were consistent with the overall efficacy rate, both suggesting that EA + CWM (98.9%) was the most effective option. For femoral BMD, the most effective for increasing BMD in the femoral neck was MO + CWM (100.0%), while ward’s triangle and trochanter were WA (95.1%). In the results of VAS in NMA, according to SUCRA values, ACE (87.9%) may be the best choice for relieving pain associated with PMOP. The optimal treatment modality indicated by each outcome indicator was not identical. The results of adverse events depicted that the side effects of acupuncture-related therapies were mild and largely self-resolving. Since the optimal treatment modality suggested by each outcome index differs. In clinical practice, physicians need to integrate their diagnostic and therapeutic experiences to identify and select the most appropriate treatment. Traditional Chinese medicine hypothesizes that kidney essence and liver-kidney deficiency are the basic pathogenesis for PMOP (70). The treatment principle should focus on tonifying the liver and kidney to strengthen bones and tendons. The selection of acupoints and the method of administration are critical factors in ensuring that acupuncture has a positive effect on the patient. Our research indicated that Shenshu (BL23), Zusanli (ST36), Ganshu (BL18), Guanyuan (RN4), Pishu (BL20), and Xuanzhong (GB39) were the most frequently utilized in different acupuncture-related therapies. However, in the MO, from DU2 to DU14 on the Governor was the most popular. These high-frequency acupuncture points are related to the meridians of the liver, spleen, and kidney meridians.

Acupuncture-related therapy has long been linked to the regulation of homeostasis (Yin/Yang) within the body. Many previous studies elucidated the mechanisms underlying acupuncture-related therapies for the treatment of PMOP. It is believed that these therapies help balance the body and restore its physiological functions by targeting specific acupuncture points. Additionally, they can increase the uterine index, elevate serum estrogen levels, and modulate the HPA axis function in the ovariectomized (OVX) rat model (71, 72). EA is a method of acupuncture that integrates traditional acupuncture with electrical impulses to enhance the stimulation of specific acupoints, which can increase BMD in PMOP rats by elevating levels of insulin-like growth factor (IGF-I and IGF-BP1) and modulating the Wnt-*β*-catenin signaling pathway (73, 74). MO has the function of harmonizing qi and blood, supporting the positive and dispelling the evil, and activating the meridians. Clinical studies have demonstrated its effectiveness in reducing lower back pain and improving bone density (75). WA is a combination of acupuncture and moxibustion, in which needles are inserted into acupuncture points, while a lit moxa stick is placed on the handle of each needle to provide simultaneous warm stimulation (22). ACE, a specialized form of acupuncture therapy, can create a continuous needling effect by inserting catgut at specific acupuncture points (76). ACE can alleviate lipid peroxidation, restore glucose homeostasis, and partial reversion of the OVX-altered amino acid metabolism to improve menopausal syndrome (77). These approaches are frequently employed in both research and therapeutic practice and are among the best treatments for PMOP in our research. Therefore, in this study, we used network meta-analysis to compare the effectiveness of commonly used acupuncture treatments and to rank their efficacy. Our findings may offer a valuable clinical reference for future investigations into the use of acupuncture in treating PMOP.

This study has several limitations: (1) The quality of the original studies is a concern, as all included studies were domestic single-center investigations, lacking multicenter research. (2) The site and nature of pain were not categorized in detail in the original studies included, and a more specific description of pain with clinical practice needs to be considered in subsequent studies. (3) There are inherent limitations associated with acupuncture therapy itself. Due to the challenges in the acupuncture procedure and the variability in patients’ sensations, achieving blinding for both patients and practitioners is particularly difficult. In acupuncture practices such as MO, ACE, and WA, the selection of acupuncture points, depth of insertion, frequency of stimulation, and retention time of needles are not clearly defined, leading to potential implementation bias. (4) Some acupuncture therapies, such as EA and WA, were included in fewer studies, which may increase the chances and reduce the reliability of the results. Furthermore, most of the original studies did not report adverse effects, preventing a comprehensive evaluation of safety. (5) While all control groups received CWM, variations in specific drugs, dosages, and treatment durations were noted. We aggregated these pharmacological approaches rather than stratifying them by individual regimens.

This study indicates that different acupuncture-related therapies may be advantageous for different outcome indicators of PMOP, potentially due to the unique characteristics of each therapy. In the future, we should consider establishing multicenter clinical trials for these therapies, standardizing methodologies, and creating a multicenter efficacy registry system utilizing blockchain technology. By integrating the results of clinical trials, we can enhance research on the underlying mechanisms, allowing different acupuncture-related therapies to leverage their strengths. The aim is to elevate acupuncture-related therapies from adjunct therapies to a central component of the precision treatment system for PMOP, thereby fostering innovation in the integration of traditional Chinese medicine and Western medicine within the bone health management paradigm.

## 5 Conclusion

The effectiveness of acupuncture-related therapies for PMOP has been systematically evaluated in this NMA. This study demonstrated that acupuncture-related therapies are superior in treating PMOP. Furthermore, EA + CWM emerged as the most effective intervention in terms of the overall clinical effectiveness rate. EA + CWM, MO + CWM, and WA all showed advantages in improving BMD. ACE ranked highest as the optimal choice for the improvement of VAS. However, potential heterogeneity among studies and acupuncture-related interventions due to the number of included studies, and sample size, resulted in some of the comparisons failing to achieve the required level of statistical significance. It is hoped that this study will provide some references for future clinical studies on different acupuncture-related therapies for the treatment of PMOP. Therefore, more multicenter, large-sample, randomized controlled clinical trials with appropriate design and methodology are necessary in the future to further validate the efficacy of different acupuncture therapies. It is hoped to further improve the mechanism study based on the clinical results and to standardize and precise the treatment of different clinical symptoms of PMOP by acupuncture-related therapies in combination with artificial intelligence technology to enhance the treatment response rate. Upgrading acupuncture-related therapies from PMOP complementary alternative therapies to the core module of the precision treatment system gives full play to the advantages of different therapies.

## Data Availability

The datasets presented in this study can be found in online repositories. The names of the repository/repositories and accession number(s) can be found here: doi: 10.6084/m9.figshare.28705172.

## Footnotes

^1^ https://www.crd.york.ac.uk/prospero/#record
