# Supplementary material for: Efficacy of acupuncture-related therapy for postmenopausal osteoporosis: a systematic review and network meta-analysis based on randomized controlled trials
Source: Front Med (Lausanne). 2025 Apr 9;12:1483819. doi: 10.3389/fmed.2025.1483819 (PMC12015985; doi:10.3389/fmed.2025.1483819)

**Appendix 2.** Eight library search strategies and results

1. Pubmed

| **search strategy：**  Search: **(((((((((((((((((Acupuncture Therapy[MeSH Terms]) OR (Acupuncture Treatment[Title/Abstract])) OR (Treatment, Acupuncture[Title/Abstract])) OR (Therapy, Acupuncture[Title/Abstract])) OR (Pharmacoacupuncture Treatment[Title/Abstract])) OR (Acupuncture Moxibustion Therapy[Title/Abstract])) OR (Acupuncture[Title/Abstract])) OR (Electroacupuncture[Title/Abstract])) OR (catgut implantation at acupoint[Title/Abstract])) OR (acupoint thread embedding therapy[Title/Abstract])) OR (acupoint thread embedding[Title/Abstract])) OR (acupoint embedding therapy[Title/Abstract])) OR (moxibustion[Title/Abstract])) OR (Acupoint sticking therapy[Title/Abstract])) OR (Needle[Title/Abstract])) OR (Ear Acupuncture[Title/Abstract]) AND (2002:2023[pdat])) AND (((((((((((((((((Osteoporosis, Postmenopausal[MeSH Terms]) OR (Perimenopausal Bone Loss[Title/Abstract])) OR (Bone Loss, Postmenopausal[Title/Abstract])) OR (Bone Losses, Postmenopausal[Title/Abstract])) OR (Postmenopausal Bone Losses[Title/Abstract])) OR (Osteoporosis, Post-Menopausal[Title/Abstract])) OR (Osteoporoses, Post-Menopausal[Title/Abstract])) OR (Osteoporosis, Post Menopausal[Title/Abstract])) OR (Post-Menopausal Osteoporoses[Title/Abstract])) OR (Post-Menopausal Osteoporosis[Title/Abstract])) OR (Postmenopausal Osteoporosis[Title/Abstract])) OR (Osteoporoses, Postmenopausal[Title/Abstract])) OR (Postmenopausal Osteoporoses[Title/Abstract])) OR (Bone Loss, Perimenopausal[Title/Abstract])) OR (Bone Losses, Perimenopausal[Title/Abstract])) OR (Perimenopausal Bone Losses[Title/Abstract])) OR (Postmenopausal Bone Loss[Title/Abstract]))) AND (((Randomized Controlled Trial[Title/Abstract]) OR (RCT[Title/Abstract])) OR (Randomized Controlled[Title/Abstract]))** |
| --- |

**
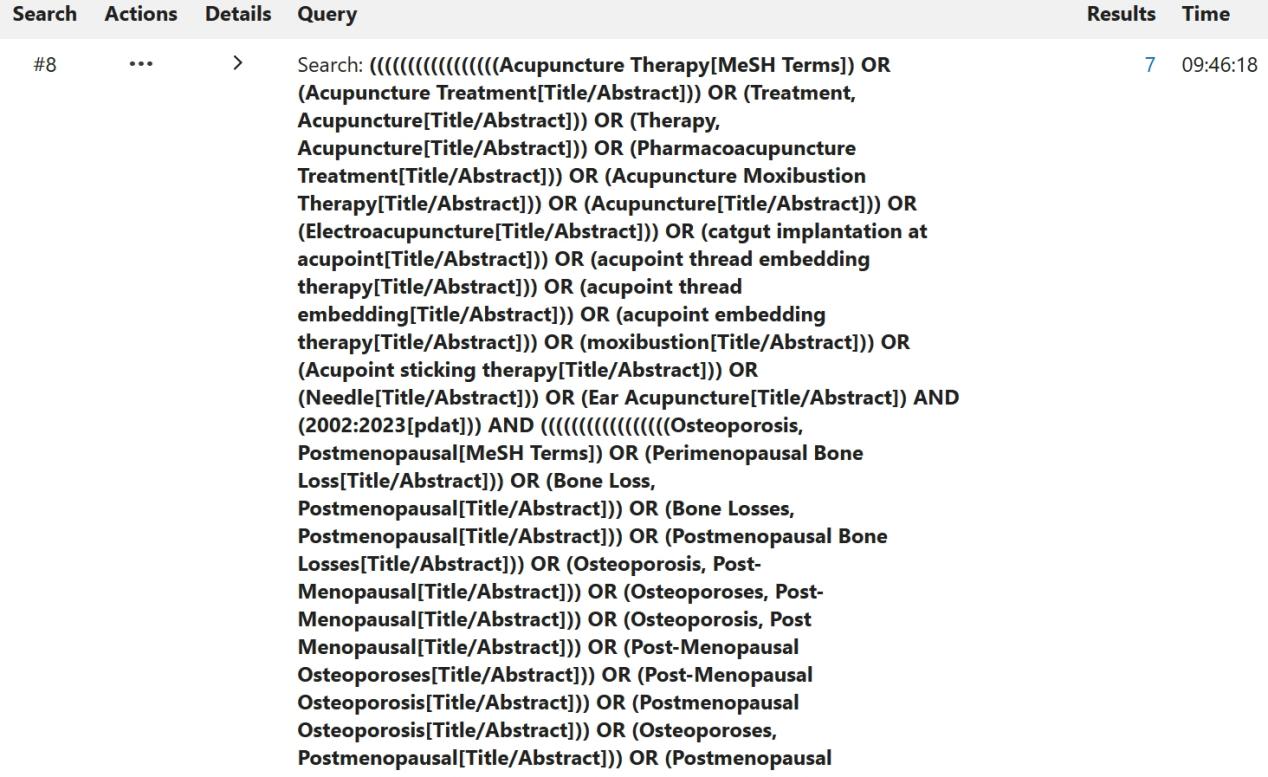
**

1.
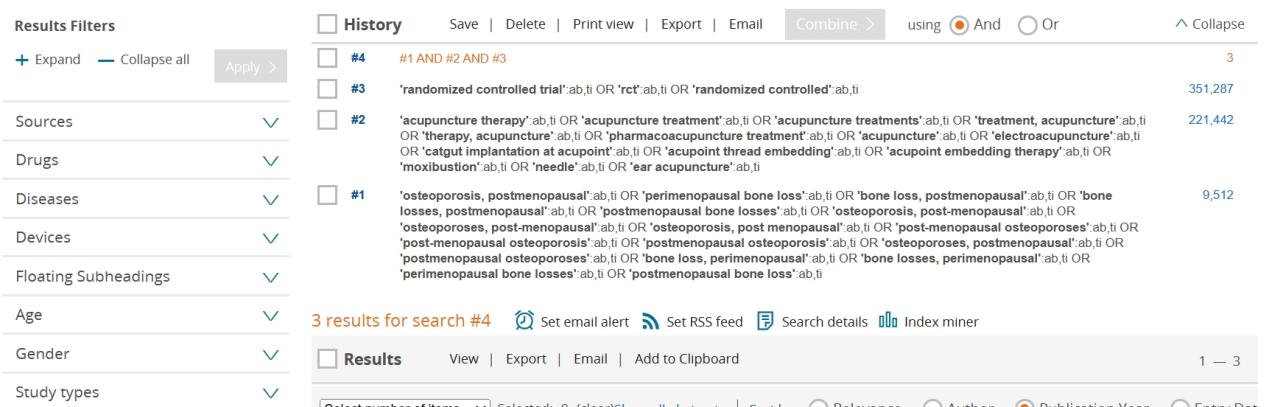
EMBASE
2.
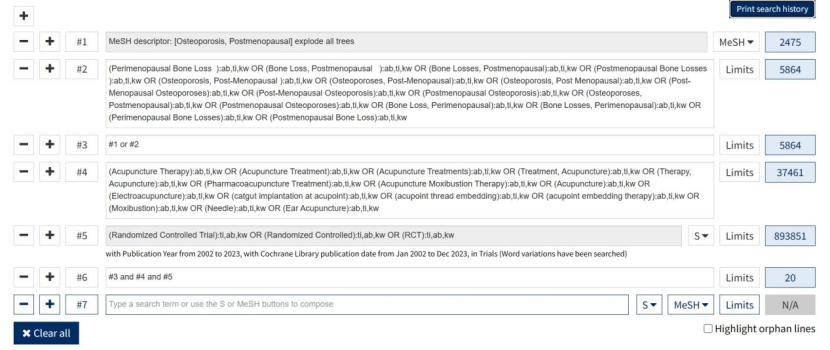
Cochrane Library
3.
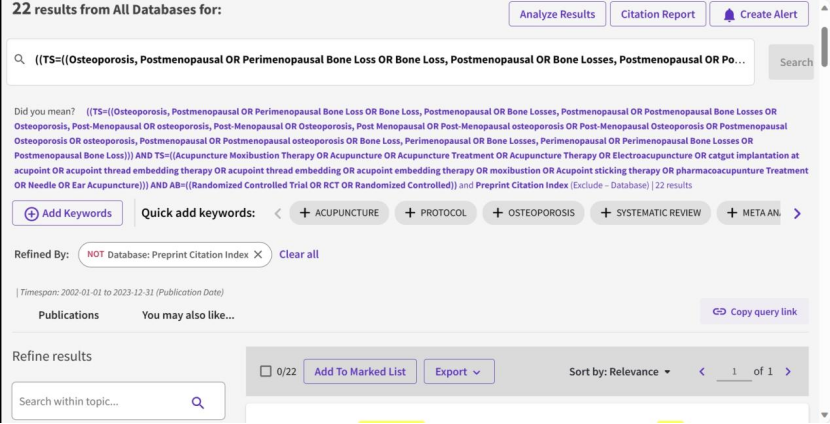
Web of science
4. China National Knowledge Infrastructure (CNKI)

SU=('骨质疏松，绝经后'+'绝经期骨丢失'+'骨质丢失，绝经后'+'绝经后骨质疏松'+'更年期骨质疏松'+'围绝经期骨质疏松'+'闭经后骨质疏松'+'绝经后骨质丢失') and SU=('针'+'针灸疗法'+'针刺'+'电针'+'穴位埋线'+'温针灸'+'艾灸'+'穴位贴敷'+'耳针') and TKA=('随机对照试验'+'随机对照'+'随机'+'随机分配'+'RCT')


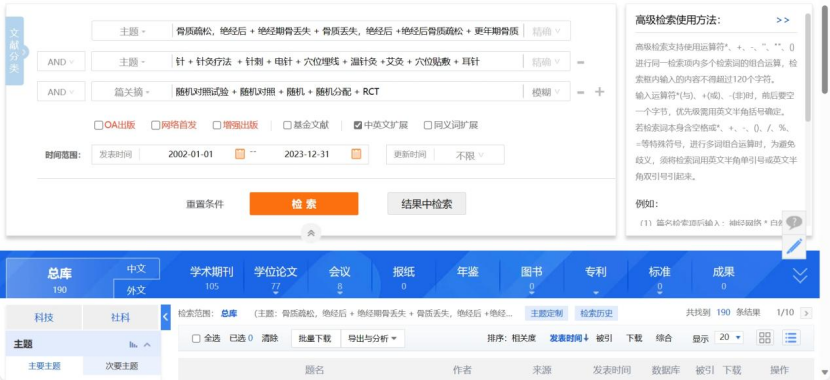


1. Wanfang Data


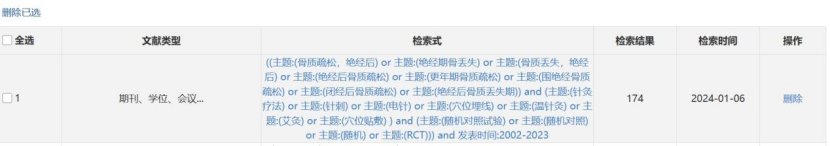


1. Chinese Scientific Journal Database (VIP)


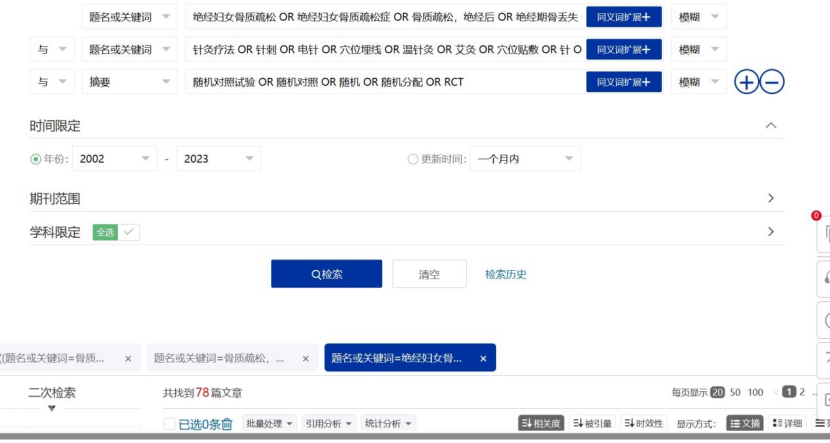

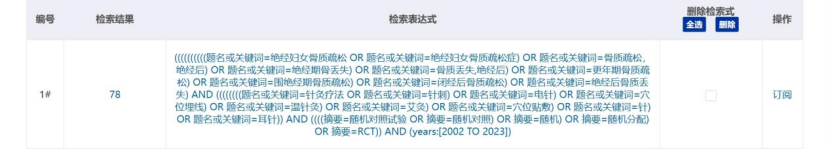


1. China Biology Medicine (CBM)


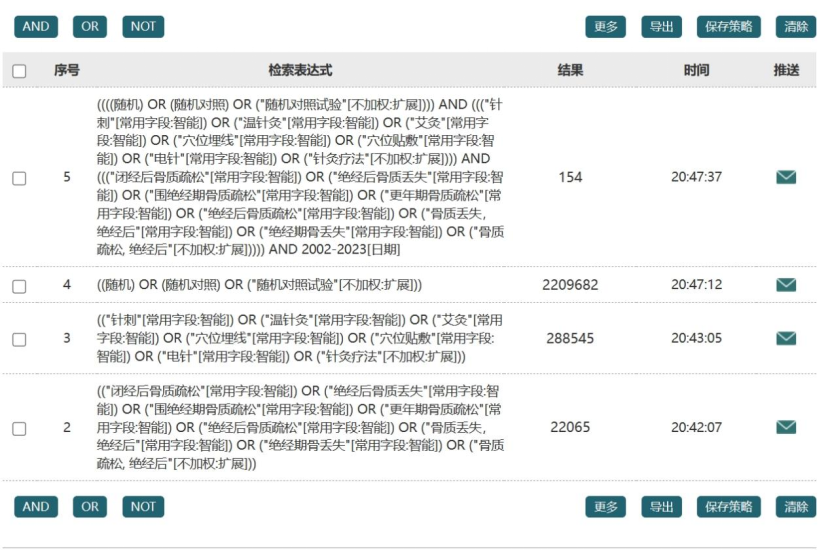

Supplement: Supplementary file 2 [file Table_2.docx]
