# Supplementary material for: Efficacy of acupuncture-related therapy for postmenopausal osteoporosis: a systematic review and network meta-analysis based on randomized controlled trials
Source: Front Med (Lausanne). 2025 Apr 9;12:1483819. doi: 10.3389/fmed.2025.1483819 (PMC12015985; doi:10.3389/fmed.2025.1483819)
Supplement: Supplementary file 3 [file Table_3.docx]

**Point-by-point responses** (**ID: 1483819**)

12-Mar-2025

Dear Editor and Reviewers,

On behalf of my co-authors, I would like to express our sincere appreciation to editor and all reviewers for their constructive comments concerning our article entitled ***‘Efficacy of acupuncture-related therapy for postmenopausal osteoporosis: A systematic review and network meta-analysis based on randomized controlled trials [ID: 1483819]’***. According to the editor and reviewers’ comments, we have made modifications to our manuscript. In this revised version, changes to our manuscript were all highlighted within the document by using red-colored text. Point-by-point responses to the editor and reviewers are listed below this letter. (Note: Page numbers in the“Manuscript - marked copy”PDF file prevail)

1. Grammar, Spelling, and Language Clarity
   Findings:
   The manuscript is generally well-written, but there are instances of awkward phrasing and inconsistent use of tenses.
   Occasional misuse of technical terminology.
   Recommendations:
   Conduct a thorough proofreading session focusing on grammar consistency, particularly in the methods and results sections.
   Simplify complex sentences to enhance readability.
   Standardize technical terms throughout the manuscript.

**Response:** We sincerely appreciate the valuable comments. We have carefully revised the grammar, spelling and clarity of language throughout the text based on your comments. (For example, Page 7, Lines 169-189)

2. Logical Structure and Content Coherence
Findings:
The abstract outlines objectives, methods, results, and conclusions effectively but could be more concise.
The introduction lacks a clearly stated hypothesis and research gap.
Some section transitions are abrupt.
Recommendations:
Streamline the abstract by removing redundant information.
Clearly state the research hypothesis at the end of the introduction.
Use appropriate transitional sentences to improve section flow.
**Response:** Thanks for your kind suggestions. We have revised the entire text in the light of your suggestions, in particular the introduction. (Page 2-4, Lines 48-91)

3. Data Presentation and Interpretation
Findings:
Figures and tables are informative but lack comprehensive captions and consistent labeling.
Statistical methods are appropriate, though interpretation of some results requires cautious discussion.
Recommendations:
Enhance figure legends and table titles for clarity.
Provide clear definitions for all abbreviations in tables.
Discuss borderline P-values and potential limitations in the discussion section.

**Response:** Thanks for your pointing this out. We have further refined the information of figures, tables and methodology sections. (Page 7-8, Lines 169-196; Page 26-27, Lines 743-793)

4. Clarity and Consistency in Tables and Figures
Findings:
Figures are inadequately labeled, and some tables lack necessary explanations.
Recommendations:
Include clear axis labels, units, and detailed captions for all figures.
Provide full definitions for all abbreviations in tables.
**Response:** Thanks for your kind comment. We have supplemented the information in the tables and figure in detail for clarity and consistency. (Page 26-27, Lines 743-793; Table1 and Figures)

5. Research Methods Detail
Findings:
Methods are described well but lack detail regarding quality control in data collection.
Statistical analysis descriptions need more clarity regarding model assumptions.
Recommendations:
Add detailed descriptions of data validation and quality control procedures.
Clarify how assumptions for statistical models were assessed and validated.
**Response:** We sincerely appreciate the valuable comments. We have added more details of the research methodology based on your suggestions. (Page 7-8, Lines 171-196)

6. References Formatting and Completeness
Findings:
References are relevant but inconsistently formatted.
Missing DOI information for some citations.
Recommendations:
Ensure all references follow the target journal's formatting style.
Include DOI information for all references where available.

**Response:** Thank you very much for your detailed review. It is a great pity that some of our Chinese references do not have DOI numbers, which leads to the lack of DOI information in some of the citations.

7. Strength of Discussion and Conclusion
Findings:
The discussion adequately links findings to existing literature but lacks emphasis on the study's unique contributions.
The conclusion summarizes key points but needs more focus on limitations and future research directions.
Recommendations:
Highlight how findings provide new insights into the field.
Discuss study limitations in detail and recommend future research directions.

**Response:** Thanks for your kind suggestions. This further elevates the meaning of our article, and I've expanded on the discussion and conclusion section based on your comments. (Page 14-18, Lines 352-471)
